# Optimizing Coronary Computed Tomography Angiography Using a Novel Deep Learning-Based Algorithm

**DOI:** 10.1007/s10278-024-01033-w

**Published:** 2024-03-04

**Authors:** H. J. H. Dreesen, C. Stroszczynski, M. M. Lell

**Affiliations:** 1 Department of Radiology, University Regensburg, Franz-Josef-Strauss Allee 11, 93053 Regensburg, Germany; 2 Department of Radiology, Neuroradiology and Nuclear Medicine, Klinikum Nürnberg, Paracelsus Medical University, Nuremberg, Germany

**Keywords:** Coronary computed tomography angiography, Single-source computed tomography, 64-Detector row computed tomography, Motion artifact reduction, Deep learning-based algorithm, Motion correction algorithm

## Abstract

Coronary computed tomography angiography (CCTA) is an essential part of the diagnosis of chronic coronary syndrome (CCS) in patients with low-to-intermediate pre-test probability. The minimum technical requirement is 64-row multidetector CT (64-MDCT), which is still frequently used, although it is prone to motion artifacts because of its limited temporal resolution and z-coverage. In this study, we evaluate the potential of a deep-learning-based motion correction algorithm (MCA) to eliminate these motion artifacts. 124 64-MDCT-acquired CCTA examinations with at least minor motion artifacts were included. Images were reconstructed using a conventional reconstruction algorithm (CA) and a MCA. Image quality (IQ), according to a 5-point Likert score, was evaluated per-segment, per-artery, and per-patient and was correlated with potentially disturbing factors (heart rate (HR), intra-cycle HR changes, BMI, age, and sex). Comparison was done by Wilcoxon-Signed-Rank test, and correlation by Spearman’s Rho. Per-patient, insufficient IQ decreased by 5.26%, and sufficient IQ increased by 9.66% with MCA. Per-artery, insufficient IQ of the right coronary artery (RCA) decreased by 18.18%, and sufficient IQ increased by 27.27%. Per-segment, insufficient IQ in segments 1 and 2 decreased by 11.51% and 24.78%, respectively, and sufficient IQ increased by 10.62% and 18.58%, respectively. Total artifacts per-artery decreased in the RCA from 3.11 ± 1.65 to 2.26 ± 1.52. HR dependence of RCA IQ decreased to intermediate correlation in images with MCA reconstruction. The applied MCA improves the IQ of 64-MDCT-acquired images and reduces the influence of HR on IQ, increasing 64-MDCT validity in the diagnosis of CCS.

**Supplementary Information:**

The online version contains supplementary material available at 10.1007/s10278-024-01033-w.

## Introduction

The European Society of Cardiology (ESC) recommends coronary computed tomography angiography (CCTA) as the diagnostic method of choice for patients with suspected chronic coronary syndrome (CCS) with a low to intermediate pre-test probability (PTP) [1]. Currently, 64-row multidetector single-source CT (64-MDCT) is considered the minimum requirement for proper CCTA imaging [2]. The 64-MDCT systems have been shown to be a valid and accurate diagnostic tool, even when compared to ≥ 128-MDCT or dual-source CT (DSCT) [3, 4]. In addition, 64-MDCT is widely available, making it an indispensable diagnostic tool in patients with CCS [1, 5, 6]. Although 64-MDCT can provide perfect images under optimal conditions, its limited temporal resolution makes it susceptible to motion artifacts in patients with high or variable heart rates (HR), especially in the right coronary artery (RCA) [1, 7, 8]. Several approaches have been proposed to reduce motion artifacts in 64-MDCT, both in terms of hardware modification (gantry rotation time, half scan rotation, high-pitch imaging, prospective (PGI) and retrospective electrocardiographic (ECG)-gated imaging) and HR control (beta-blockers or ivabradine) [3, 4, 8, 9]. However, these approaches have limitations either due to physical limits or contraindications [9, 10]. For further image enhancement, novel software-based approaches in the form of motion correction algorithms (MCA) offer a suitable solution for motion-disturbed images.

Several MCA based on different technical approaches have been introduced in the last decade [11]. However, only a few MCA have proven their clinical utility and are commercially available [12, 13]. Furthermore, the clinical applicability of most of these MCA is limited mainly because of either vendor-specificity, high effective dose, poor performance at high or irregular HR, or long computation time [11, 14–16]. The latest MCA variants are based on deep-learning networks [11]. In several phantom trials and small patient studies, they have shown remarkable results in improving the image quality (IQ) of motion-impaired images in an acceptable computation time [11, 14, 17]. However, clinical data for these deep learning-based MCA are still scarce. The aim of this study was to evaluate the performance of a recently introduced deep learning-based MCA (*Deep PAMoCo*) on IQ in a large set of real-world patient CCTA data sets and to demonstrate the potential clinical utility of this MCA [15].

## Materials and Methods

### Image Data, Algorithm, and CT-Scanning

124 CCTA data sets of consecutive patients scanned with the same 64-MDCT system and the same CT protocol were retrieved from the Picture Archiving and Communication System and included in this study. The clinical indication for CCTA was according to clinical guidelines [2]. Original image data were anonymized, and patients are not identifiable. Consecutive patient data in which at least one vascular segment was affected by motion artifacts were selected for the evaluation with a conventional reconstruction algorithm (CA) and the MCA. Since the MCA is applied to already reconstructed image data, no raw data is required. The MCA can, therefore, be used on different CT systems without any limitations.

The function of the applied MCA is based on partial angle reconstructions (PAR) computed with a motion vector field (MVF) generated by a Deep Neural Network (DNN). After an initial reconstruction of the CCTA images, the position of the coronary arteries is determined using a segmentation software. PAR of the coronary arteries are created from this data by forward and backprojecting data. PAR are characterized by a very high temporal resolution, virtually freezing the individual PAR. The PAR are then mapped by a MVF to the same motion state. MVF are generated by a DNN and compute a motion vector for each PAR. Finally, the motion-corrected PAR are re-inserted into the original reconstruction, resulting in a motion-compensated image. More detailed technical information about the MCA can be found elsewhere [15].

The scanning protocol included calcium-scoring, test-bolus-tracking, and CCTA. CCTA imaging was performed using a 64-MDCT (Siemens Definition 64, Siemens Healthineers, Erlangen, Germany) with a gantry rotation time of 0.33s, a collimation of 64 × 0.6mm, an automatic, weight-adjusted tube voltage between 100 and 120kVp, and automatic exposure control. Acquisition was performed with PGI. PGI was performed at a maximum HR ≤ 80 beats per minute (bpm) during an R-R interval of 60–80% in diastole (average 68%). Low-dose calcium-scoring was performed before CCTA to estimate the patient’s calcium load. A calcium score of 1000 was considered the upper limit for CCTA. Patients with a calcium score >1000 were referred to the catheter laboratory. CCTA was performed by trained staff. Beta-blockers were administered orally or i.v. if HR was ≥65bpm after checking contraindications. Sublingual nitroglycerine was administered 2–3min before the examination. For the examination, patients were placed in the supine position, head first. The field of view (FOV) was estimated considering the size of the heart (approximately from 2cm below the carina to the lower edge of the apex cordis). Contrast medium (CM; Solutrast 370, Bracco, Milan, Italy) was administered via an antecubital intravenous line at a flow rate of 6ml/s followed by 30ml of saline at the same flow rate. Body mass index (BMI), age, sex, mean HR, and intra-cycle HR changes (ΔHR) were registered.

### Image Quality Assessment

**Fig. 1:**
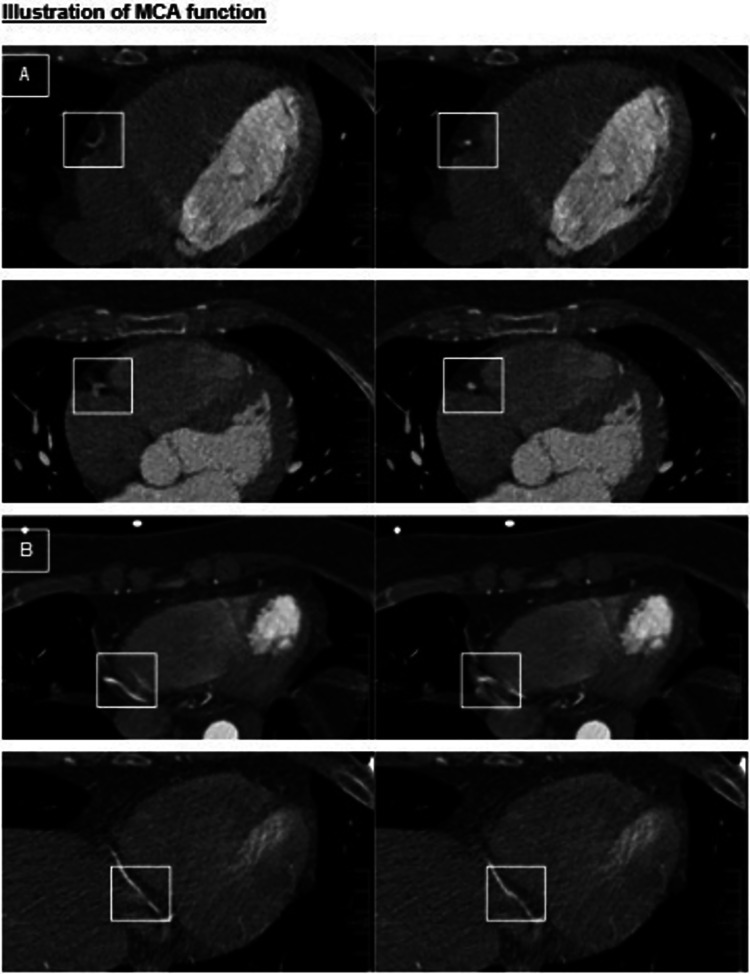
**A** Motion artifact elimination by MCA at segment 2. **B** MCA inserted artifacts at segment 3 (*n* = 11)

Images were evaluated by a radiology resident trained for the evaluation of CCTA images. IQ was assessed per-segment, per-artery (right coronary artery = RCA, left anterior descending artery = LAD, left circumflex artery = LCx), and per-patient. Per-segment assessment was performed in regard to the Society of Cardiovascular Computed Tomography-guidelines for the interpretation and reporting of CCTA [18] using a 17-segment approach. A minimal vessel diameter of 2mm was chosen for quality evaluation. IQ was determined using a 5-point Likert score in terms of image evaluability. The 5-point Likert score provides accurate information on IQ without being overwhelming. Evaluability was determined based on image readability and the amount of motion artifacts according to previous studies [19]: 1 = unacceptable; 2 = below average; 3 = average; 4 = above average; 5 = excellent (Table 1). The total amount of motion artifacts was assessed by counting the motion artifacts per-artery (RCA, LAD, LCx) by identifying typical patterns of motion artifacts as “crescents,” “tails,” and “horns” (Fig. 1A). MCA-inserted artifacts were assessed by identifying typical patterns as “steps” or vessel “duplications” (Fig. 1B).

**Table 1:**
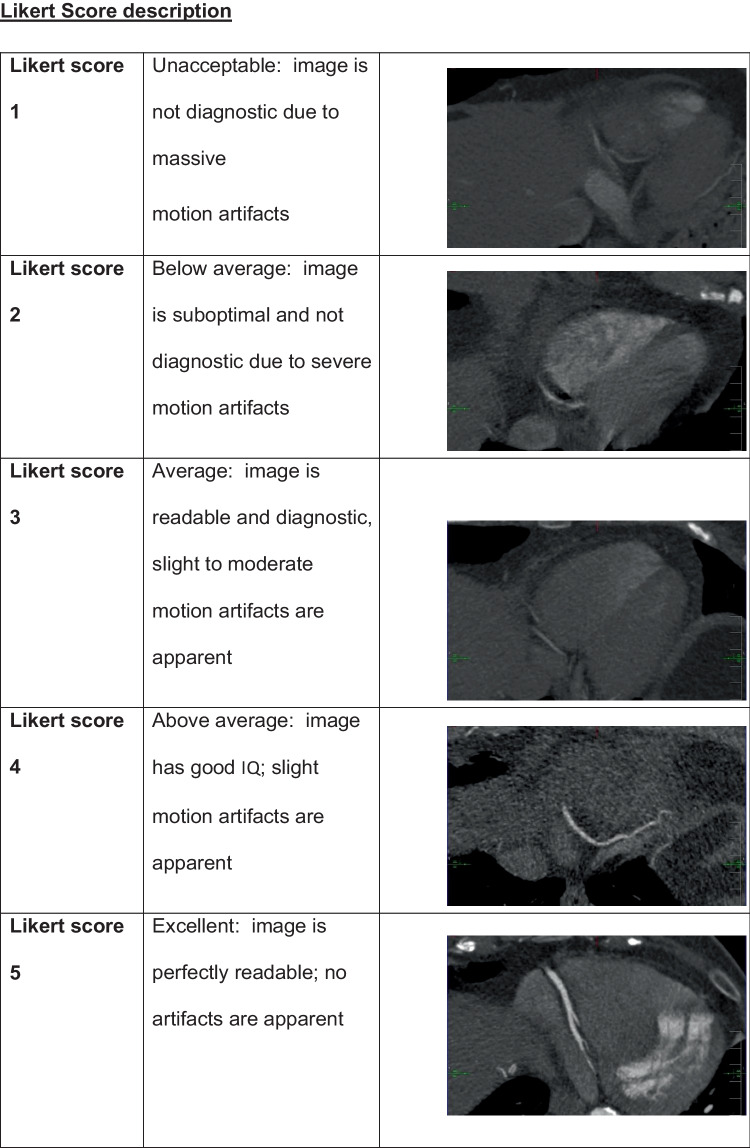
Likert score description

### Statistics

Statistical analysis was carried out with JASP team (2022). JASP (version 0.16.4) [computer software]. Continuous variables are expressed as mean ± standard deviation (SD). The central tendency of non-dichotomous categorical variables is expressed as median and percentage. Significance was tested using paired samples tests. A one-tailed *p*-value of <0.01 is considered to indicate statistical significance in IQ assessment. IQ between the CA and the MCA was compared using the Wilcoxon-Signed-Rank test for ordinal variables. Rank-Biserial correlation was chosen as the effect size measurement. Normality of continuous data was assessed by applying the Shapiro–Wilk test. As continuous data were not normally distributed, the non-parametric Wilcoxon-Signed-Rank test and Rank-Biserial correlation were applied. Correlation analysis between BMI, age, sex, mean HR, and ΔHR and IQ was performed using Spearman’s Rho. A two-tailed *p*-value of <0.01 is considered to indicate statistical significance. Graphs were created using GraphPad Prism, Prism 9 for Windows 64-bit, version 9.5.1 (733), January 26, 2023, tables were created using Microsoft® Excel® 2019 MSO (Version 2303 Build 16.0.16227.20202) 64 Bit.

## Results

**Fig. 2:**
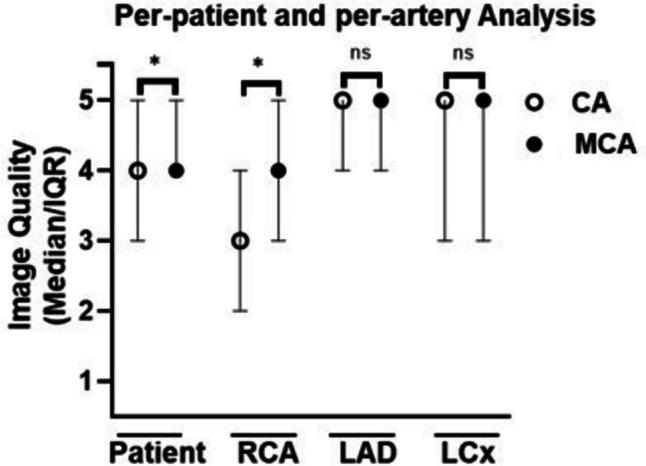
Median and interquartile range of IQ per-patient and per-artery with CA and MCA due to a 5-point Likert score. Significance is marked with an asterisk

**Fig. 3:**
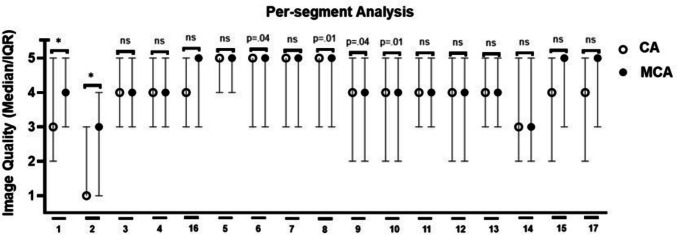
Median and interquartile range of IQ per-segment with CA and MCA due to a 5-point Likert score. Significance is marked with an asterisk

CCTA data sets of 124 patients were evaluated (Table 2). Of these, eleven data sets were excluded due to severe stack transition, vessel calcifications, and medical devices (stents and pacemakers) producing massive artifacts. BMI was missing in 20 patients; sex, age, ΔHR, and mean HR in nine patients. IQ of 113 patients, 333 arteries, and 3019 segments was evaluated (Fig. 2 and 3; Supplementary Table 1).

**Table 2:** Study population

**Study population**			
**Total (*n*)**	124		
**Male/female**	59	55	

	**Mean**	**Range**	** ± SD**
**Mean age (years)**	59,49	21–95	12,53
**Mean BMI (kg/m**^**2**^**)**	27,58	18,9–43,03	5,21
**Mean HR (bpm)**	63,92	43–133	11,88
**Mean ΔHR (bpm)**	7,14	0–105	14,16

**Fig. 4:**
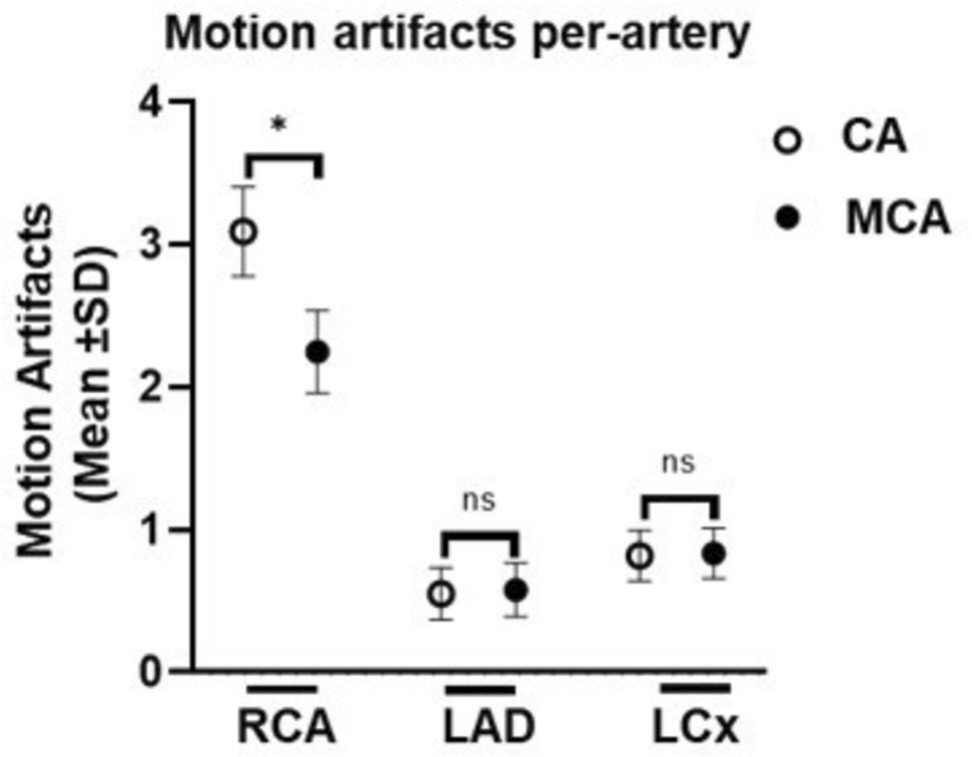
Mean ± SD of motion artifacts per-artery. Significance is marked with an asterisk

**Fig. 5:**
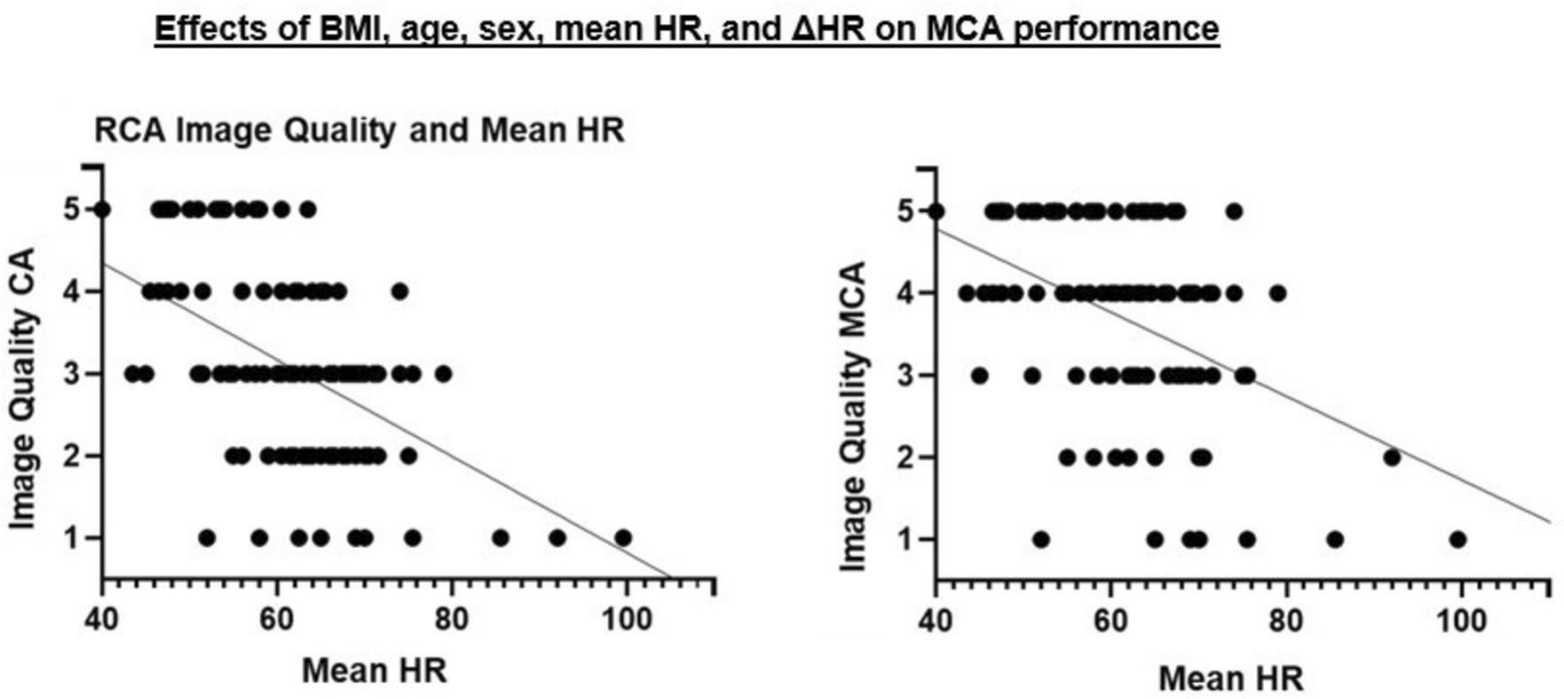
Correlation (Spearman’s Rho) of IQ improvement with CA and MCA due to a 5-point Likert score in the RCA and mean HR

Per-patient, unacceptable or below-average images decreased from 9.65% to 4.39%, and above-average or excellent images increased from 67.54% to 77.2%. Per-artery, the RCA improved significantly. Here, the percentage of unacceptable or below-average images decreased from 36.36% to 18.18%, and above-average or excellent images increased from 31.82% to 59.09%. Per-segment, RCA segments 1 and 2 benefited from the MCA. Unacceptable or below-average images decreased from 33.63% to 22.12% and from 71.68% to 46.9%, respectively, while above-average or excellent images increased from 44.25% to 54.87% and from 19.47% to 38.05%, respectively. The total number of artifacts was determined per-artery (Fig. 4; Supplementary Table 2). We observed a decrease in motion artifacts from 3.11 ± 1.65 to 2.26 ± 1.52 in the RCA. There was no significant decrease in motion artifacts in the LAD or LCx. In 11 out of 3019 segments, the IQ deteriorated due to MCA-inserted artifacts, especially in RCA segments 1 and 3. These artifacts mostly resembled vessel “duplications” or “steps”. The correlation between IQ and BMI, age, mean HR, ΔHR, and sex was tested per-artery using Spearman’s Rho (Fig. 5; Supplementary Table 3). Mean HR and IQ correlated significantly negatively in all three coronary arteries. The correlation was strong for RCA reconstructed with CA and intermediate for MCA. Correlation was weak for LAD and LCx reconstructed with both CA and MCA. There was no significant correlation between IQ and BMI, age, ΔHR, or sex.

## Discussion

In this study, we evaluated the performance of a novel deep learning-based MCA by comparing IQ of 64-MDCT-acquired CCTA images. As in previous studies, the RCA and its segments 1 and 2 were found to be most prone to motion artifacts, as these are the most motile vessel segments [7]. MCA reconstruction had the greatest effect in these segments in improving IQ and reducing the total number of motion artifacts. Baseline IQ of LAD and LCx per-artery and per-segment was initially much better; MCA-improvement of LAD and LCx was negligible. On the per-patient level, we observed an overall improvement of IQ. By evaluating potential disturbers, we found a significant negative correlation between mean HR and IQ for RCA, LAD, and LCx in CA- and MCA-reconstruction. However, the influence of mean HR was strong in the CA-reconstruction and intermediate in the MCA-reconstruction of the RCA. Correlation between mean HR and IQ of LAD and LCx was weak in both CA and MCA. BMI, age, sex, and ΔHR had no significant impact on IQ.

Recently, various MCA-based approaches have been published to mitigate motion artifacts. Two vendor-specific MCA are currently available (2023): SnapShot Freeze (SSF) 1 and its successor SSF2 (GE Healthcare, Waukasha, WI, USA) [13, 20]. In the clinical setting, SSF1 improved IQ and interpretability in ≥ 64-MDCT independent of HR and BMI [21, 22]. In addition, good IQ was maintained even at high HR, allowing wider application of PGI leading to a lower total effective dose [21, 23]. Therefore, SSF1 is considered a useful tool to assist CCTA in CCS diagnosis [12]. Positive effects of SSF2 on IQ are even more profound compared to its predecessor [13]. Unfortunately, both MCA are vendor-specific and only applicable on vendor-specific CT scanners [17]. Besides SSF1 and 2, there have been several attempts to develop even more effective and widely applicable MCA [11]. However, most of these suffer from limitations due to high effective dose, poor performance at high or irregular HR, or long computation time [11, 16, 24, 25]. The recently introduced deep learning-based MCA might be a solution. Deep learning-based MCA can be applied post-acquisitionally without the need for raw data [26]. By this, they have a very short computation time and can be used vendor-independently [11, 15]. However, larger studies on the performance of deep learning-based MCA are still scarce. Therefore, their clinical applicability cannot yet be assessed although phantom studies are promising [11, 15, 25].

In this study, we have found that the applied deep learning-based MCA *Deep PAMoCo* improves the IQ of 64-MDCT-acquired images [13, 15]. By this, the rate of non-diagnostic images and false-positive results could be remarkably reduced, especially at higher HR [22, 27, 28]. As CCTA is already considered to have a high-negative predictive value, this could further increase its validity for the diagnosis of CCS [1]. Especially regarding the limited temporal resolution of 64-MDCT, the presented MCA seems to be attractive to enhance 64-MDCT-acquired images. However, the applied MCA can also be expected to be useful in combination with high-end imaging technology, as high or irregular HR can also disturb ≥ 128-MDCT and DSCT imaging [29]. Besides IQ improvement, the tested MCA could also reduce the effective dose during CCTA, as PGI could be applied at higher HR, and by this more widely [21, 23]. However, as IQ still correlated with HR at an intermediate level, the presented MCA should be considered as a support and not as a substitute for HR control [30]. Finally, the tested MCA seems to be especially attractive in regard to its broad applicability due to its short computation time of 15s per entire CCTA image and its vendor-independent use [11, 15, 17]. Thus, the presented MCA resembles a low-effort software upgrade for CCTA imaging performed with a 64-MDCT.

This study has limitations. Firstly, since we wanted to test the ability of the MCA to compensate for motion artifacts and to improve IQ, patient data were not given in this trial. Secondly, in this study, we had to exclude eleven images completely and two partially because of stack transition, vessel calcifications, and medical devices (stents and pacemakers) producing massive artifacts. In addition, due to a lack of documentation, we were unable to determine BMI in 20 patients and mean HR, ΔHR, age, and sex in 9 patients. Thirdly, the evaluation of IQ was conducted by a sole professional. Consequently, we cannot provide an inter-observer agreement. Fourthly, the IQ assessment was conducted by employing a 5-point Likert score, consistent with previous research [19]. However, it is essential to note that there is no officially recommended approach for evaluating IQ, and therefore, the assessment lacks standardization. Consequently, the comparability with studies utilizing different assessment scores is restricted. Fifthly, the primary objective of this study was to evaluate the performance of the applied MCA in enhancing the IQ of real patient CCTA images. It is crucial to emphasize that the findings should not be generalized to other deep learning methods, given our limited study population and the focus on a sole MCA. Sixthly, this was a single-center study. We recommend further studies at other radiology centers to increase the power and validity of our findings. Moreover, as this study aimed to evaluate the impact of a deep learning-based MCA on IQ, we cannot draw conclusions regarding its clinical utility. Further research is needed to evaluate the impact of MCA on diagnostic accuracy e.g. using invasive coronary angiography as a reference. Thus, it would also be possible to evaluate the impact of vessel calcification on IQ and MCA-related effective dose reduction. Finally, we did not compare the tested MCA with vendor-specific or other MCA. Thus, we cannot determine the superiority of the presented MCA.

## Conclusion

In conclusion, this study has demonstrated on the one hand that the applied deep learning-based MCA is able to improve IQ in a large set of 64-MDCT-acquired real-patient images and, on the other hand, to reduce HR impact on IQ. Thus, the presented MCA can be considered as a promising example of deep learning-based MCA. Now, further studies should be done to evaluate the effectiveness of the presented MCA in regard to other MCA and to assess its clinical utility and diagnostic accuracy.

### Supplementary Information

Below is the link to the electronic supplementary material.

Supplementary file1 (DOCX 19 KB)

Supplementary file2 (PDF 255 KB)

## Abbreviations

BMI: Body mass index
CA: Conventional algorithm
CCTA: Coronary computed tomography angiography
CCS: Chronic coronary syndrome
DSCT: Dual source computed tomography
ECG: Electrocardiogram
ESC: European society of cardiology
FOV: Field of view
HR: Heart rate
IQ: Image quality
i.v.: Intra-venously
LAD: Left descending artery
LCx: Left circumflex artery
MCA: Motion correction algorithm
MDCT: Multidetector computed tomography
PGI: Prospective electrocardiographic-gated imaging
PTP: Pre-test probability
RBC: Rank-biserial correlation
RCA: Right coronary artery
SSF: SnapShot Freeze
ΔHR: Intra-cycle HR changes
