# Supplementary material for: Optimizing Coronary Computed Tomography Angiography Using a Novel Deep Learning-Based Algorithm
Source: J Imaging Inform Med. 2024 Mar 4;37(4):1548–56. doi: 10.1007/s10278-024-01033-w (PMC11300758; doi:10.1007/s10278-024-01033-w)
Supplement: Supplementary file 1 — Supplementary file1 (DOCX 19 KB) [file 10278_2024_1033_MOESM1_ESM.docx]

Supplementary Figure 1. Flow chart of methodology
