# Supplementary material for: Optimizing Coronary Computed Tomography Angiography Using a Novel Deep Learning-Based Algorithm
Source: J Imaging Inform Med. 2024 Mar 4;37(4):1548–56. doi: 10.1007/s10278-024-01033-w (PMC11300758; doi:10.1007/s10278-024-01033-w)
Supplement: Supplementary file 2 — Supplementary file2 (PDF 255 KB) [file 10278_2024_1033_MOESM2_ESM.pdf]

Optimizing coronary computed tomography angiography using a novel deep learning-based algorithm

Journal of digital imaging

Hendrik J. H. Dreesen, Dep. of Radiology, University Regensburg, Franz-Josef-Strauss Allee 11, 93053 Regensburg, Germany, Hendrik.dreesen@web.de, ORCID: 0009-0000-5293-695X

IQ per-patient, per-artery, and per-segment

	CA					MCA						
**IQ**	**1**	**2**	**3**	**4**	**5**	**1**	**2**	**3**	**4**	**5**	**p**	**RBC**
**Per-Patient**												
	2	9	26	48	29	2	3	21	42	46	**< .001**	-0.895
**(%)**	1,75	7,89	22,8	42,1	25,44	1,75	2,63	18,42	36,84	40,35		
**Per-Artery**												
**RCA**	11	29	35	19	16	7	13	25	37	28	**< .001**	-1.000
**(%)**	10	26,36	31,82	17,27	14,55	6,36	11,82	22,73	33,64	25,45		
**LAD**	2	2	10	33	66	2	2	11	28	70	0.175	-0.333
**(%)**	1,75	1,75	8,77	28,95	57,89	1,75	1,75	9,65	24,56	61,4		
**LCx**	3	8	18	24	57	3	7	21	21	58	0.558	0.028
**(%)**	2,7	7,21	16,22	21,62	51,35	2,7	6,31	18,92	18,92	52,25		
**Per-Segment**												
**1**	17	21	25	19	31	9	16	26	30	32	**< .001**	-0.674
**(%)**	15,04	18,58	22,12	16,81	27,43	7,96	14,16	23,01	26,55	28,32		
**2**	62	19	10	12	10	30	23	17	24	19	**< .001**	-1.000
**(%)**	54,87	16,81	8,85	10,62	8,85	26,55	20,35	15,04	21,24	16,81		
**3**	7	9	20	31	38	6	8	24	29	38	0.432	-0.043
**(%)**	6,67	8,57	19,05	29,52	36,19	5,71	7,62	22,86	27,62	36,19		
**4**	3	5	12	21	29	3	5	11	19	31	0.117	-0.600
**(%)**	4,29	7,14	17,14	30	41,43	4,35	7,25	15,94	27,54	44,93		
**16**	3	6	8	13	27	3	4	8	13	29	0.175	-0.333
**(%)**	5,26	10,53	14,04	22,81	47,37	5,26	7,02	14,04	22,81	50,88		
**5**	1	6	11	18	77	1	6	12	16	78	0.579	0.000
**(%)**	0,88	5,31	9,73	15,93	68,14	0,88	5,31	10,62	14,16	69,03		
**6**	1	5	26	14	67	1	5	24	14	69	0.036	-1.000
**(%)**	0,88	4,42	23,01	12,39	59,29	0,88	4,42	21,24	12,39	61,06		
**7**	0	12	24	13	64	0	12	25	10	66	0.383	-0.200
**(%)**	0	10,62	21,24	11,5	56,64	0	10,62	22,12	8,85	58,41		
**8**	0	18	21	16	58	0	16	22	14	61	**0.010**	-1.000
**(%)**	0	15,93	18,58	14,16	51,33	0	14,16	19,47	12,39	53,98		
**9**	9	19	19	8	49	9	18	18	7	51	0.036	-1.000
**(%)**	8,65	18,27	18,27	7,69	47,12	8,74	17,48	17,48	6,8	49,51		
**10**	9	28	12	8	44	9	26	11	7	48	**0.007**	-1.000
**(%)**	8,91	27,72	11,88	7,92	43,56	8,91	25,74	10,89	6,93	47,52		
**11**	4	17	26	10	54	4	17	26	10	53	0.500	-1.000
**(%)**	3,6	15,32	23,42	9,01	48,65	3,64	15,45	23,64	9,09	48,18		
**12**	15	9	11	7	31	15	9	11	5	33	0.173	-1.000

**(%)**	20,55	12,33	15,07	9,59	42,47	20,55	12,33	15,07	6,85	45,21		
**13**	5	19	18	17	44	5	17	20	17	44	0.286	-0.333
**(%)**	4,85	18,45	17,48	16,5	42,72	4,85	16,5	19,42	16,5	42,72		
**14**	15	11	9	2	31	15	11	9	1	32	0.500	-1.000
**(%)**	22,06	16,18	13,24	2,94	45,59	22,06	16,18	13,24	1,47	47,06		
**15**	0	0	4	1	3	0	0	4	1	3	0.500	-1.000
**(%)**	0	0	50	12,5	37,5	0	0	50	12,5	37,5		
**17**	1	8	3	5	15	1	6	4	4	16	0.173	-1.000
**(%)**	3,13	25	9,38	15,63	46,88	3,23	19,35	12,9	12,9	51,61		

Supplementary Table 1 IQ per-patient, per-artery, and per-segment due to a five-point Likert scale. Significant results in bold. CA = conventional algorithm, MCA = motion correction algorithm, IQ = Image quality, p = p-value, RBC = Rank-biserial correlation.

Motion artifacts and MCA inserted artifacts

	Motion artifacts						MCA inserted artifacts
	**CA (Mean)**	**Range**	**±SD**	**MCA (Mean)**	**Range**	**±SD**	**n**
**RCA**	3,11	0-7	1,65	2,26	0-6	1,52	11
**LAD**	0,55	0-5	0,98	0,58	0-4	1,02	0
**LCx**	0,81	0-3	0,94	0,83	0-3	0,94	0

Supplementary Table 2 Mean ±SD of motion artifacts and total number (n) of MCA inserted artifacts per-artery. CA = Conventional algorithm, MCA = Motion correction algorithm, SD = Standard deviation, RCA = Right coronary artery, LAD = Left descending artery, LCx = Left circumflex artery.

Effects of BMI, age, sex, mean HR, and ΔHR on MCA performance

	CA			MCA		
	**RCA**	**LAD**	**LCx**	**RCA**	**LAD**	**LCx**
**Gender**						
Spearmans Rho	0.165	-0.003	-0.121	0.167	-0.061	-0.113
p	0.123	0.977	0.263	0.119	0.572	0.294
**Age**						
Spearmans Rho	2.366×10^{-4}	-0.063	0.032	0.016	-0.057	0.019
p	0.998	0.562	0.766	0.880	0.601	0.864
**R-R**						
Spearmans Rho	-0.117	-0.116	-0.078	-0.038	-0.121	-0.088
p	0.279	0.281	0.470	0.727	0.263	0.415
**Mean HR**						
Spearmans Rho	-0.510	-0.295	-0.287	-0.403	-0.289	-0.328
p	**< .001**	**0.005**	**0.007**	**< .001**	**0.006**	**0.002**
**ΔHR**						
Spearmans Rho	0.049	-0.110	-0.083	0.018	-0.155	-0.096
p	0.650	0.306	0.441	0.865	0.149	0.376
**BMI**						
	-0.095	-0.082	-0.062	0.022	-0.028	-0.046
	0.379	0.447	0.566	0.839	0.797	0.668

Supplementary Table 3 Correlation (Spearmans Rho and p-value) of improvement per-artery and mean HR, ΔHR, BMI, age, and sex. Significant results in bold. CA = Conventional algorithm, MCA = Motion correction algorithm, RCA = Right coronary artery, LAD = Left descending artery, LCx = Left circumflex artery, p = p-value, HR = Heart rate, ΔHR = intra-cycle HR change, BMI = body mass index.
